# Oral Administration of *Ganoderma lucidum* to Lead-Exposed Rats Protects Erythrocytes against Hemolysis: Implicates to Anti-Anemia

**DOI:** 10.1155/2015/463703

**Published:** 2015-08-02

**Authors:** Shahdat Hossain, Sujan Bhowmick, Saiful Islam, Liza Rozario, Sabrin Jahan, Mehedi Hassan, Marzan Sarkar, Bazlul Karim Choudhury, Sohel Ahmed, Hussain Shahjalal

**Affiliations:** ^1^Department of Biochemistry and Molecular Biology, Laboratory of Alternative Medicine and Behavioral Neurosciences, Jahangirnagar University, Savar, Dhaka 1342, Bangladesh; ^2^Manikganj Medical College, Manikganj, Dhaka 1800, Bangladesh

## Abstract

We studied the effect of chronic oral exposure to lead acetate (PbA) on the sensitivity of RBC to hemolysis and whether the sensitivity could be decreased by feeding the rats with extract of medicinal mushroom *Ganoderma lucidum*. Three groups of rats, control, PbA-exposed, and *G. lucidum (Gl)+PbA,* were used. PbA (3 mM) was administered via drinking water and *G. lucidum* extract by gavage at 300 mg/Kg BW/day for 12 weeks. Afterwards, the rats were killed and washed RBCs were subjected to hemolysis in the presence of Fenton's reagents. Hemolysis was determined by estimating the amount of released hemoglobin. The levels of lipid peroxide (LPO) and GSH were determined from RBC membranes and whole RBCs, respectively. The levels of TNF*α* and LPO also were determined from hepatic tissues. The RBCs of PbA-exposed rats displayed significantly higher sensitivity to hemolysis than those of the *Gl*+PbA rats. The levels of LPO increased and GSH decreased in the RBCs, with concomitant increases in the levels of hepatic TNF*α* and LPO in the PbA-exposed rats. The degree of hemolysis was significantly low in the RBCs of *Gl*+PbA rats, concurrently with amelioration of hepatic parameters. Finally, the study suggests that PbA-induced-hemolysis and related oxidative-toxicity might be minimized by consumption of *G. lucidum*.

## 1. Introduction

Red blood cell (RBC) primarily transports oxygen throughout the body. In doing so, the RBC has to compromise with both endogenous and exogenous sources of reactive oxygen species (ROS) that damage and deteriorate its function. However, the RBC has an innate defense mechanism, comprising glutathione and a host of antioxidative enzymes [1, 2] to avoid its tremendous wear and tear. Because short life span RBCs are constantly undergoing turnover and making the blood system highly sensitive to environmentally very poisonous elements such as lead (Pb), the investigation on the effect of Pb is of special significance in Bangladesh because of the fact that Pb poisoning has been one of the major alarming public-health problems in Bangladesh [3]. RBC serves as the initial receptacle of absorbed Pb and distributes Pb throughout the body, making it available to other tissues. Pb interferes with normal red blood cell formation by inhibiting important enzymes. In addition, Pb damages red blood cell membranes and interferes with cell metabolism, thus shortening the survival of each individual cell [4–6]. Approximately 99% of the Pb in blood is associated with red blood cells; the remaining 1% resides in blood plasma [7, 8]. On the one hand, erythrocytes detoxify numerous circulatory xenobiotics [9], many of which directly confer oxidative insults to the erythrocytes [10]; on the other hand, the erythrocytes themselves are highly vulnerable to oxidative stress because of the high contents of oxygen and polyunsaturated fatty acids in the membrane [11]. All of these factors make the RBCs very much susceptible to hemolysis. One of the aims of the Pb poisoning-related investigations should thus be the reduction of susceptibility of RBCs to hemolysis that ultimately leads to hemolytic anemia.

*Ganoderma lucidum* is a medicinal mushroom and used in traditional Chinese medicine, with a very broad spectrum of biological activities and pharmacological functions [12].* G. lucidum* is reportedly known to have anticancer, antitumor, antidiabetic, and anti-inflammatory effects [13–15]. Exposure to Pb can cause hypochromic microcytic anemia [16]. This may relate to the fact that Pb is absorbed by iron-absorbing machinery, confers competitive inhibition, and interferes with heme biosynthesis [17]. Anemia in children leads to increased morbidity and mortality [18]. Because children may be exposed to levels of Pb which could adversely affect their health without exhibiting clinical symptoms, it is vital to adopt a preventive approach. Therefore searching for agents capable of reducing the levels of Pb from the body could be considered as one of the important preventive measures. Very recently, we have reported that the oral administration of* G. lucidum* extract prevents paracetamol-induced hepatotoxicity in rats [19] and erythrocyte hemolysis in rats [20], suggesting oral administration of this medicinal mushroom extract can play, at least partially, role in the reduction of Pb-induced hemolysis and related anemia. In this study, we thus investigated whether the chronic administration of Pb results in an increased sensitivity of erythrocyte to hemolysis and, if so, then whether the oral administration of the extract of* G. lucidum* decreases it. Also, the liver is the first organ that encounters the absorbed Pb. Dead erythrocytes are also recycled in the liver, thus releasing all their (RBCs') contents including toxic Pb in this organ. Therefore, the mechanisms of action of the antihemolytic effect of* G. lucidum* and that of the effect of Pb on the hepatic tissues were discussed.

## 2. Materials and Methods

### 2.1. Chemicals

Lead acetate was used as test chemicals in the present study. Lead acetate (PbA) of AR grade was procured from E Merck. TEP (1,1,3,3-tetraethoxypropane), reduced glutathione (GSH), DTNB [5,5′-dithiobis (2-nitrobenzoic acid)] were purchased from Sigma Aldrich. TNF*α* was from Santa Cruz Biotechnology, CA, USA. Horseradish peroxidase-conjugated anti-rabbit secondary antibody was from Cell Signaling Technology. The primary antibody anti-rabbit TNF*α* was from Santa Cruz Biotechnology, CA, USA. ELISA grade BSA was from Sigma Aldrich. Tetramethylbenzidine is from (Invitrogen) Life Technologies, USA. All other chemicals were of analytical grade.

### 2.2. Animals

**Figure 1:**
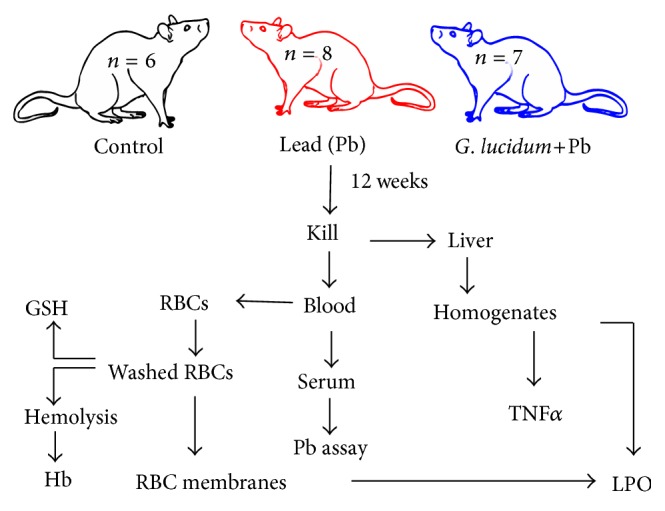
Experimental design. RBCs: red blood cells; GSH: reduced glutathione; Hb: hemoglobin; TNF*α*: tumor necrosis factor *α*.

Wistar rats obtained from animal breeding colony of the icddr,b, Dhaka, were used in the present study. The animals were fed on standard pellet diet and maintained under controlled laboratory conditions (12 h light: 12 h dark; temperature 25 ± 2°C; relative humidity 50 ± 10%). The inbred second generation rats (15 weeks old, 180–200 g body weight, [BW]) were randomly divided into three groups: the control group (*n* = 6), lead acetate- (PbA-) exposed group (*n* = 8), and* G. lucidum-*fed PbA-exposed group (*Gl*+PbA, *n* = 7) (Figure 1). The PbA-exposed group was orally fed PbA at 3 mM prepared drinking water. The extract of* G. lucidum* dissolved in distilled water was orally administered at 300 mg/Kg BW/day. The control group was orally fed a similar volume of the dH_2_O alone. Oral administration of PbA and/or* G. lucidum was* continued for 12 weeks. The rats were cared for and killed in accordance with the guidelines of laboratory animals and approved by the Institutional Animal Ethical Committee at Jahangirnagar University, Savar, Dhaka, Bangladesh.

### 2.3. RBC Preparation

RBCs were prepared as previously described by Hashimoto et al. (2015) [21]. After deep anesthesia with pentobarbital blood from individual rat was collected from inferior vena cavae with heparinized syringe. Half of the blood was used for plasma collection and the other half was mixed with Locke's solution (154 mM NaCl, 5.6 mM KCl, 2.3 mM CaCl_2_, 1 mM MgCl_2_, 3.6 mM NaHCO_3_, 5 mM glucose, and 5 mM HEPES; pH 7.2) and pelleted at 300 ×g for 10 minutes in plastic tubes. The supernatant was discarded and the RBCs were washed thrice by using the same Locke buffer solution. The buffy coat and a portion of the upper layer of the RBCs were removed in each wash. The remaining RBCs were immediately subjected to hemolysis and intracellular GSH assay or used for preparation erythrocyte ghost membranes. RBCs were counted by Sysmex XS-1000i.

### 2.4. Hemolysis Assay

RBC suspensions [10^7^ cells/mL Locke's solution (154 mM NaCl, 5.6 mM KCl, 2.3 mM CaCl_2_, 1 mM MgCl_2_, 3.6 mM NaHCO_3_, 5 mM glucose, and 5 mM HEPES; pH 7.2)] from each rat were subjected to incubation for 1 h at 37°C with freshly prepared Fenton's reagents [H_2_O_2_ (45 mM) + FeSO_4_ (2 mM)]. Then, RBCs were pelleted down by centrifuging the samples at 300 ×g for 10 min. The supernatant was aspirated and the extent of hemolysis was quantified by determining the amounts of released hemoglobin (Hb) into the supernatant at 540 nm against Hb standard.

### 2.5. Antihemolytic Effect of *α*-Tocopherol

RBCs [10^7^ cells/mL Locke's solution (154 mM NaCl, 5.6 mM KCl, 2.3 mM CaCl_2_, 1 mM MgCl_2_, 3.6 mM NaHCO_3_, 5 mM glucose, and 5 mM HEPES; pH 7.2)] from the Pb-nonexposed rats were subjected to Fenton's reagents [H_2_O_2_ (45 mM) + FeSO_4_ (2 mM)] in the absence or presence of *α*-tocopherol (0–100 *μ*M). After 1 h of incubation at 37°C, the extent of hemolysis was determined with hemoglobin standard, as described above.

### 2.6. Erythrocyte Reduced Glutathione (GSH) Assay

Erythrocyte GSH was measured according to the method of Moron et al. [22]. A part of the washed RBCs was suspended in ice-cold dH_2_O containing 20 mM EDTA. After a brief sonication, the suspension was immediately treated with 10% trichloroacetic acid (final concentration) and vortexed for 10 min. Afterwards, the contents were centrifuged at 10000 ×g for 30 min. Following centrifugation, 100 *μ*L of the supernatant was mixed with 0.4 M tris buffer (pH 8.9). The whole solution was mixed well and 10 mM DTNB was added and the absorbance was read within 5 min of addition of DTNB at 412 nm against reagent blank with no homogenate. For blank reading, the homogenate was substituted with distilled water. The amount of glutathione in the erythrocytes was expressed as pmol of GSH/10^7^ RBCs.

### 2.7. Preparation of RBC Ghost Membranes

Washed RBCs were suspended in 40 volumes of ice-cold 5 mM Tris-HCl buffer (pH 7.0), containing 1 mM EDTA, and centrifuged (10000 ×g, 60 minutes, 4°C). Supernatant was discarded and washing was repeated until the erythrocyte membranes appeared whitish. The membranes were stored at −80°C.

### 2.8. Preparation of Liver Homogenates

After drawing blood, the liver from each rat was separated, perfused with ice-cold saline. Afterwards, a 10% liver tissue homogenate was prepared with phosphate buffer (100 mM, pH 7.4) containing 1% phenylmethylsulfonyl fluoride (PMSF). The homogenate was centrifuged at 1000 ×g to remove unruptured tissues and debris and the resultant homogenate was assigned as whole homogenate. A part of the whole homogenate was further spun for 1 h at 10000 ×g to prepare cytosolic fraction to measure TNF*α* in the liver tissues. The samples were stored at −20°C until analysis.

### 2.9. Lipid Peroxide (LPO) of RBC Ghost Membranes and Liver Tissues

The basal levels of lipid peroxide (LPO) in the RBC membranes and hepatic tissues were determined by estimating the thiobarbituric acid reactive substances (TBARs), as described previously [23]. The RBC membranes or liver tissue whole homogenates (0.1 mL) from each of the rats were added to 0.1 mL of 8.1% (w/v) sodium dodecyl sulphate, 2 mL of 0.4% thiobarbituric acid in 20% acetic acid (pH 3.5), and 0.1 mL distilled water. Each tube was tightly capped and heated at 95°C for 1 h. After cooling the tubes with tap water, 2 mL of n-butanol-pyridine (15 : 1, v/v) was added and shaken vigorously for about 10 minutes. The tubes were then centrifuged at 1200 ×g for 10 minutes at room temperature (digital centrifuge; DSC-1512SD). The absorbance of the upper organic layer was measured at 532 nm. TEP (1,1,3,3-tetraethoxypropane) was utilized as standard.

### 2.10. ELISA for Liver Tumor Necrosis Factor Alpha (TNF*α*)

The multiwell plate was coated with liver cytosolic fraction in 0.1 M sodium bicarbonate, pH 9.6 at 4°C overnight, and then blocked with 1% BSA in tris-buffered saline (TBS). The primary antibody anti-rabbit TNF*α* (Santa Cruz Biotechnology, CA, USA) at 1 : 1000 dilutions was incubated in the plate for overnight period at 4°C. Horseradish peroxidase-coupled anti-rabbit IgG (Biosource International, Inc., Camarillo, CA, USA) was used as the secondary antibody and incubated for 2 h at room temperature before the addition of tetramethylbenzidine (Invitrogen, Life Technologies, USA) substrate to develop color. The reaction was stopped by addition of 0.1 N HCl after incubation for 30 min at room temperature. Well coated with only 0.1 M carbonate buffer, pH 9.6 was used as blank. The plates were analyzed with a multiwell plate reader (Erba Lisascan II, Mannheim, Germany) at 450 nm.

### 2.11. Analysis of Serum Pb by Atomic Absorption Spectrophotometer

The serum from each rat was allowed to dry at 120°C until reaching a constant weight and concentrated nitric acid and hydrogen peroxide (1 : 1 v/v) were added. The digestion flasks were heated to 1300°C until all the materials were dissolved and diluted with double dH_2_O appropriately. The element lead was assayed using Varian 240 Atomic Absorption Spectrophotometer. The results were expressed as *μ*M.

### 2.12. *In Vitro* Antioxidative Potential of* G. lucidum* Extract

Evaluation of* in vitro* antioxidant activity of* G. lucidum* extract was performed by determining the (i) DPPH-free radical scavenging ability and (ii) anti-LPO ability, as previously described from this laboratory [24, 25]. For the determination of* in vitro* anti-LPO ability of the extract, homogenates of RBC membranes from nonexposed rats were divided into control membranes (*n* = 5), oxidative stress-induced (OS, Fenton's reagents-incubated) membranes, and OS+*G. lucidum* extract-treated membranes. The sample mixtures were then incubated at room temperature for 4 hours. Afterwards, the levels of lipid peroxide (LPO) were determined, as indicator of oxidative stress, following the methods of Hossain et al. (2011; 2004) [26, 27]. LPO of RBC membranes was calculated as nmol/mg of protein.

### 2.13. Other* In Vitro* Methods

Total polyphenols and total flavonoids of the* G. lucidum* extract were measured as previously described [24]. Liver-function-specific enzymes such as aspartate aminotransferase (AST) and alanine aminotransferase (ALT) were measured by using enzymatic reagent kits, as previously described [20].

Total protein was measured by bicinchoninic acid (BCA) method.

### 2.14. Statistical Analysis

All the data were expressed as mean ± SE (standard error of mean). The significance of difference in means among different groups was determined by one-way analysis of variance (ANOVA), followed by Fisher's PLSD test for post hoc comparisons by using GraphPad prism software version 5.0. *P* < 0.05 was considered statistically significant.

## 3. Results

### 3.1. Effect of Preadministration of* G. lucidum* on Fenton's Reagents-Induced Hemolysis of RBCs

**Figure 2:**
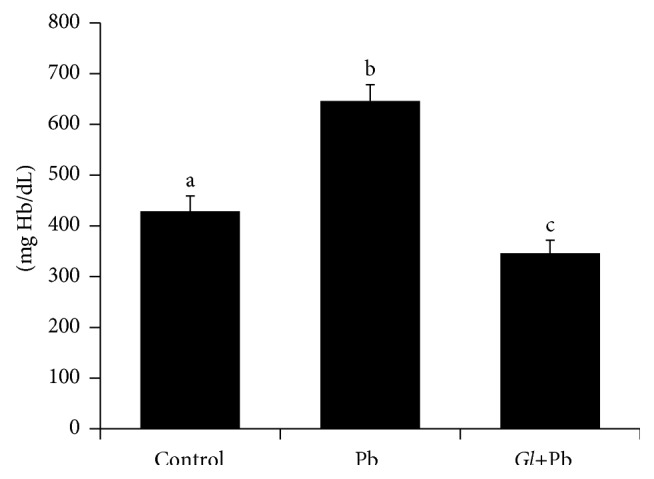
Effect of Pb on hemolysis. Results are mean ± SE (*n* = 6 ~ 8), each with duplicate determinations. Bars with different alphabets are significantly different at *P* < 0.05. Data were analyzed with one-way ANOVA, with Fisher's PLSD for post hoc comparison.

The susceptibility of RBCs to the oxidative stress-induced (Fenton's reagents) hemolysis was measured in all erythrocyte samples of the controls, PbA-exposed and* G. lucidum*+PbA-fed rats (Figure 2). The RBCs of the PbA-exposed rats displayed the highest sensitivity to Fenton's reagents-instigated hemolysis. It was ~51% higher (*P* < 0.05), as compared to that of the control. However, the oral administration of* G. lucidum* extract to the PbA-fed rats significantly reduced the degree of hemolysis in the rats (*G. lucidum*+PbA) (Figure 2).

**Figure 3:**
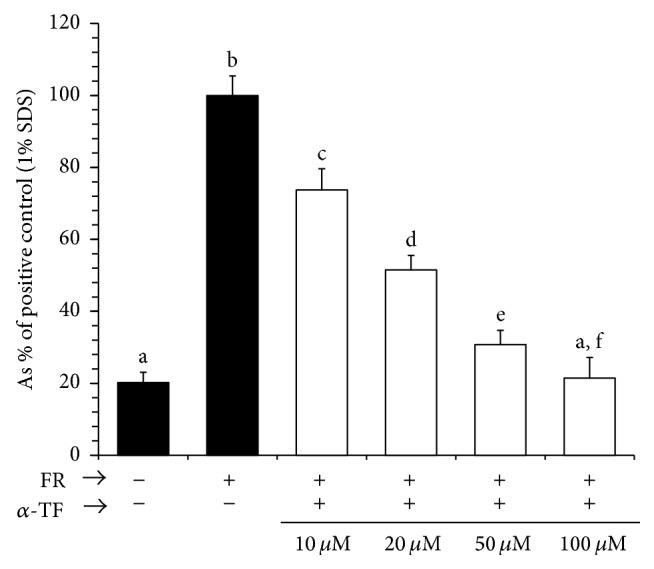
Effect of *α*-tocopherol on oxidative stress (FR: Fenton's reagents) induced hemolysis. Results are mean ± SE (*n* = 6 ~ 8). Bars with different alphabets are significantly different at *P* < 0.05. *α*-tocopherol (*α*-TF) dose-dependently inhibited the degree of hemolysis, as indicated by the gradual decreases in the levels of released hemoglobin. 1% SDS was used as positive control. Data were analyzed with one-way ANOVA, with Fisher's PLSD for post hoc comparison. (−) indicates absence. (+) indicates presence.

To understand whether the antioxidative defense was involved in lowering the extent of hemolysis, the RBCs from the non-Pb exposed rats were subjected to the oxidative stress by Fenton's reagents in the absence (0 *μ*M) or presence of *α*-tocopherol (10–100 *μ*M). 1% SDS was used as positive control of hemolysis (Figure 3). Hemolysis data were normalized to those of the positive control. The levels of hemolysis were decreased with increases in the concentrations *α*-tocopherol in the samples, as indicated by the decreased amount of released hemoglobin in the buffer (Figure 3).

### 3.2. Effect of Oral Administration of* G. lucidum* on Intracellular Erythrocyte GSH

**Figure 4:**
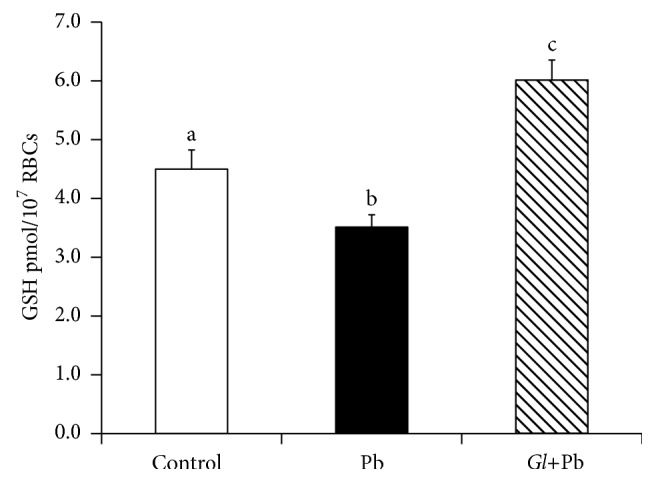
Effect of oral administration of* G. lucidum* on the intracellular GSH levels of erythrocytes. Results are mean ± SE (*n* = 6), each with duplicate determinations. Bars with different alphabets are significantly different at *P* < 0.05. Data were analyzed with one-way ANOVA, with Fisher's PLSD for post hoc comparison.

The oral PbA-exposure caused a significant decrease in the levels of GSH in the RBCs of PbA-exposed rats; however, it was significantly increased in the extract-fed PbA-exposed (PbA+*G. lucidum*) rats (Figure 4).

### 3.3. Effects of Oral Administration of* G. lucidum* on the Basal Levels of LPO in the RBC Membranes and Hepatic Tissues

**Figure 5:**
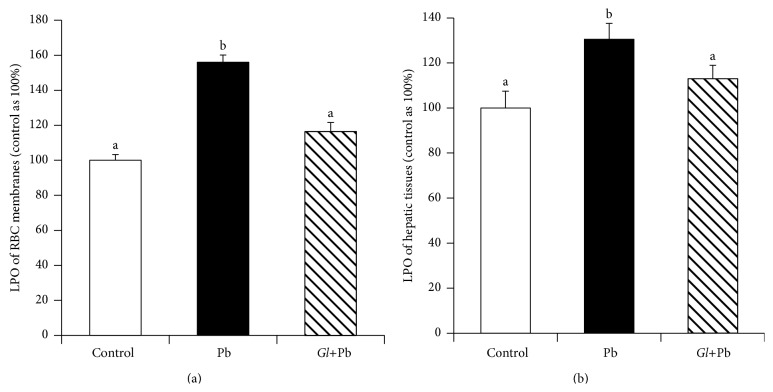
Effects of oral administration of* G. lucidum* on the levels of lipid peroxide (LPO) of RBC membranes and hepatic tissues. Results are mean ± SE (*n* = 6), each with duplicate determinations. Bars with different alphabets are significantly different at *P* < 0.05. Data were analyzed with one-way ANOVA, with Fisher's PLSD for post hoc comparison.

The levels of LPO were significantly increased (by 55%) in the RBC membranes of the Pb-exposed rats, as compared to those of the RBCs of control rats. The oral administration of the rats with* G. lucidum*, however, significantly decreased the levels of LPO, as compared to that of the PbA-exposed rats (Figure 5(a)). The levels of LPO were also significantly increased in the hepatic tissues of PbA-exposed rats (Figure 5(b)). The oral administration of* G. lucidum* to the rats, however, reduced the levels to those of the controls (Figure 5(b)).

### 3.4. Effects of Oral Administration of* G. lucidum* on Hepatic Tumor Necrosis Factor *α* (TNF*α*)

**Figure 6:**
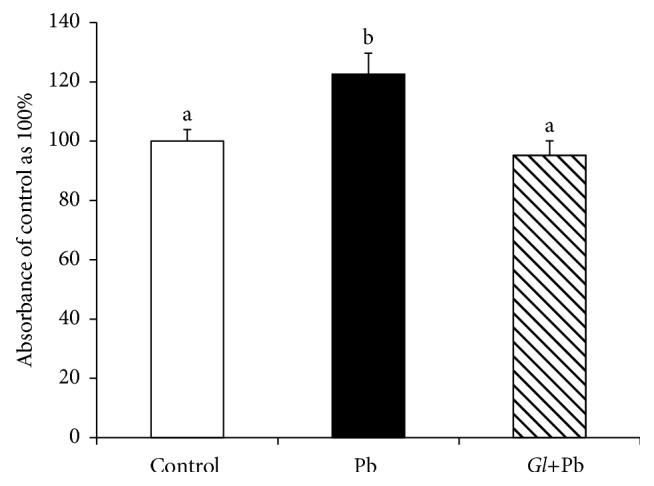
Effect of oral administration of* G. lucidum* on the hepatic levels of tumor necrosis factor *α* (TNF*α*). Results are mean ± SE (*n* = 6 ~ 8), each with duplicate determinations. Bars with different alphabets are significantly different at *P* < 0.05. Data were analyzed with one-way ANOVA, with Fisher's PLSD for post hoc comparison.

The levels of TNF*α* were significantly augmented in the liver tissues of the PbA-exposed rats, as compared to those of the controls; however, it was significantly reduced in the* G. lucidum*+PbA-administered rats (Figure 6).

### 3.5. Effects of* G. lucidum* Extract on the Plasma Pb Levels

The basal Pb levels in the plasma of the control rats were 0.95 ± 0.03 *μ*M. The plasma levels of Pb rose (by >85%) to >1.82 ± 0.05 *μ*M in the PbA-exposed rats, while it dropped significantly (*P* < 0.05) in the plasma of the* G. lucidum*+PbA rats (1.43 ± 0.03 *μ*M).

### 3.6. Evaluation of Antioxidative Potentials of* G. lucidum* Extract

The extract of the* G. lucidum* had considerable amounts of antioxidant phytochemicals, such as total polyphenols (~60.6 mg gallic acid equivalent/g extract) and total flavonoids (~10 mg catechin equivalent/gm extract). The* G. lucidum* extract possessed significant DPPH-radical scavenging activity [IC_50_, concentration required to scavenge 50% of 0.4 mM of DPPH, of* G. lucidum* was 26 *μ*g/mL extract and that of the vitamin C was 24 *μ*g/mL]. Incubation of RBC membrane samples with Fenton's reagents significantly stimulated the oxidative stress (OS) in the membranes, as indicated by the increased levels of lipid peroxide (LPO). The OS-induced increases in the levels of LPO, however, were repressed in the presence of the* G. lucidum* extract, thus demonstrating a strong anti-LPO ability of this extract [LPO_nmol/mg  protein_ (or in percent): control = 24 ± 1.5 (100%); OS = 56 ± 2.5 (235%); OS+*G. lucidum* = 22 ± 1.5 (127%)]. These antioxidative properties of the extract thus suggest a potential therapeutic efficacy of* G. lucidum* extract to protect against oxidative stress. These propositions are further supported by the fact that the oral Pb exposure significantly increased the plasma levels of hepatic structural integrity-related enzyme markers, including ALT and AST. However, the levels of these enzymes were significantly ameliorated upon administration of* G. lucidum* extract (ALT: control, 36.5 ± 3.5; PbA, 48.5 ± 4.45;* Gl*+PbA, 37.4 ± 4.9 U/L) (AST: control, 99.9 ± 10; PbA, 121 ± 9.7;* Gl*+PbA, 66 ± 8.5 U/L).

## 4. Discussion

In the present study, we demonstrate that Pb increases susceptibility of erythrocytes to hemolysis and that oral administration of* G. lucidum* reduces the susceptibility to hemolysis. The underlying mechanism(s) of the heightened sensitivity to hemolysis of the RBCs of the Pb-exposed rats may relate to the fact that Pb-exposure made their erythrocytes more vulnerable to oxidative stress, which ultimately brought about the leakage of the bilayer membranes and finally caused an increased release of hemoglobin in the presence of oxidant (Fenton's reagents). Notably, the hydroxyl radical is able to penetrate deep into the lipid head groups region [28, 29] and may initiate chain reactions in the bilayer membranes. Interestingly, the oral administration of* G. lucidum* extract significantly reduced the oxidative stress and hence the extent of hemolysis. This finding led us to infer that the presence of antioxidants in the extract of* G. lucidum* might have conferred the oxidative defense against hemolysis by Fenton's reagents. To support the proposition that antioxidants had protected the RBCs against hemolysis, we collected RBCs from nonexposed rats, washed them, and subjected them to oxidative stress (by hydroxyl radicals of Fenton's reagents) in the presence of natural antioxidant *α*-tocopherol (10, 20, 50, and 100 *μ*M). As expected, *α*-tocopherol dose-dependently inhibited the oxidative stress-induced hemolysis, thus demonstrating that the reduction in the degree of hemolysis of the RBCs of the* G. lucidum*+Pb rats might have, at least partially, been occurring due to the presence of antioxidant-like substances in the* G. lucidum* extract and/or building up of an antioxidative defense in their RBCs. De Rosa et al. (1954) [30] were the first to report the protection by vitamin E against anemia due to Pb toxicity. Cassi et al. (1972) [31] also reported that vitamin E deficiency displays increased propensity for hemolysis and anemia. Thus our results of inhibition of hemolysis in the presence of *α*-tocopherol are consistent with these studies. Pb induces two types of anemia: acute high-level Pb poisoning has been associated with hemolytic anemia; in chronic Pb poisoning, Pb induces anemia by both interfering with erythropoiesis and diminishing red blood cell survival [32]. Pb inhibits several enzymes that are critical to the synthesis of heme. It should be emphasized, however, that anemia is not an early manifestation of Pb poisoning and is evident only when the blood Pb level is significantly elevated for prolonged periods. Yet, numerous studies have reported that Pb poisoning is associated with hemolytic anemia [33, 34]. Both the antioxidant and the thiobarbituric acid experiments suggested that RBC hemolysis may be related to lipid peroxidation. Although Pb is not a transition metal, the catalysis of peroxidative reactions by Pb may be a major contributor to the toxic effects of this metal [35]. Dose- and time-dependent increases in peroxides in hepatic microsomal membranes occurred in response to Pb [36]. GSH is the single most powerful antioxidant in our body. The erythrocyte GSH plays a vital role in mitigating the damaging effects of reactive oxygen species (ROS) encountered in the circulation [37] and produced by continuous oxidation of hemoglobin within the cytosol of the erythrocyte [38, 39]. The whole body acquires GSH supply primarily through the RBCs; therefore, intracellular GSH levels were determined from the whole RBCs. Pb decreased the levels of intracellular GSH. Thus the decreased GSH levels in the Pb-exposed RBCs might have resulted from the intracellular adaptive response (utilization) of the RBCs to ameliorate Pb toxicity. Interestingly, the intracellular GSH levels of RBCs were increased by the oral administration of* G. lucidum*. We again consider that the antioxidants present in the* G. lucidum* might have battled with the free radicals derived from oxidative stress; thus the intracellular resources of GSH remained relatively high and hence played ameliorating roles against hemolysis. Our results are consistent with the reports of Omobowale et al. (2014), where Pd decreased the levels of GSH [40]. The reduction of the levels of GSH in the RBCs is thus consistent with the rises of the levels of LPO in the Pb-exposed rats. The increases in the levels of LPO in our Pd-exposed rats are thus directly indicating that Pd-poisoning induces a dropping down of the antioxidative defense and leads to hemolysis. Gugliotta et al. (2012) [41] reported that the exposure of erythrocytes to Pb leads to a reduction in the average lifetime of the erythrocytes and the subsequent development of anemia. Though the mechanism(s) is not clearly known, one important effect of lead toxicity in erythrocytes consists of increasing intracellular calcium [Ca^2+^](i), which in turn causes alterations in cell shape and volume and it is associated with cellular rigidity, hemolysis, senescence, and apoptosis [42]. Whatever the mechanism is consistent with the above reports, we provide evidence that Pb increases sensitivity of RBC to hemolysis, leading to anemia, and that the oral administration of* G. lucidum *reduces the sensitivity to hemolysis.

Practically, the liver, via the portal vein, is exposed to enterally absorbed Pb and it is the tissue that shows the largest repository of lead (33%) followed by the kidney cortex and medulla [43]. Pb induces oxidation in hepatic microsomal membranes [36]. Pb also causes increased expression of TNF*α* in the liver tissues [44]. This leads us to determine the levels of LPO, as indicator of Pb-induced oxidative stress, and proinflammatory TNF*α*, in the liver tissues. The increased levels of LPO and/or TNF*α* in the Pb-exposed rats are thus consistent with these reports. Again, the oral administration of the* G. lucidum* significantly ameliorated these cell-damaging effects of Pb in the liver.

## 5. Conclusion

Recently, the farming and commercialization of* G. lucidum* have been started by different private entrepreneurs in Bangladesh with the assistance of its agricultural extension programs. Herbal remedies are relatively effective, cheap, and almost devoid of side effects, compared to synthetic agents [45]. These findings thus point to the fact that complementary herbal therapies could be a good choice here in Bangladesh, where 26% of her 150-million population still live under poverty line [46] and iron deficiency affects its ~50% of all children and ~70% of all women [47]. Iron deficiency is the most common cause of anemia in Bangladesh [48]. Hence the attractive therapeutic strategies focusing on the modulation of Pb-induced toxicity are warranted. Very likely with arsenic toxicity, the lead toxicity has already posed severe health problems in Bangladesh. Anemia is one of the most well-known toxic health effects associated with Pb exposure. Various mechanisms have been suggested for Pb-associated anemia including interference with iron transport, shortening of erythrocyte life span, and inhibition of the globulin synthesis and the impairment of heme metabolism [49, 50]. Our study clearly indicates that oral administration of* G. lucidum* extract significantly ameliorates the hemolysis, with concurrent inhibitions of oxidative stress of the RBC membranes and liver tissues. Finally we suggest that consumption of the* G. lucidum *could be used as one of the prophylactic means to reduce the body-burden of toxic Pb and Pb-toxicity-related early hemolysis and hence to combat anemia. Further studies are underway to understand the exact mechanism of action of* G. lucidum* on Pb-induced toxicity.
